# Mummy berry pseudosclerotia survive for several years

**DOI:** 10.3389/fpls.2023.1214369

**Published:** 2023-09-11

**Authors:** J. W. Pscheidt, J. P. Bassinette, B. Warneke

**Affiliations:** Department of Botany & Plant Pathology, Oregon State University, Corvallis, OR, United States

**Keywords:** *Monilinia vaccinii-corymbosi*, blueberry, plant disease, apothecia, pseudosclerotia

## Abstract

Organic blueberry production in the PNW has many challenges, including diseases like mummy berry caused by the fungus *Monilinia vaccinii-corymbosi* (Reade) Honey. Management recommendations focus on reducing overwintering pseudosclerotia, however, it is unknown how long they survive. Based on qualitative observations pseudosclerotia are hypothesized to survive multiple years after contact with the soil surface. The development of apothecia from *M. vaccinii-corymbosi* pseudosclerotia was evaluated over multiple years at a location without a history of blueberry production. A total of 1,000 pseudosclerotia were placed on field soil plots in 2018 and replicated eight times. Another 100 pseudosclerotia were placed in wire corrals on field soil and replicated fifteen times. Plots and corrals were regularly examined each spring for the emergence of apothecia. The pseudosclerotia were able to survive, germinate, and produce apothecia for up to five years after their placement. Very few pseudosclerotia produced apothecia in any year, varying from 0 to 18 at any observed time. Pieces of partial or whole pseudosclerotia were observed for up to three years after placement. Our study shows that a pseudosclerotial “seed bank” exists under blueberry bushes, necessitating a long-term implementation of mummy berry cultural management tactics.

## 1 Introduction

Blueberries are an important agricultural commodity in the Pacific Northwest (PNW), and the area accounts for 48% of the total production of highbush blueberries (*Vaccinium corymbosum* L.) in the US and 60% of the total organic production (Strik et al., 2019). Oregon produced 145 million pounds of blueberries in 2021, with an annual economic impact of more than $375 million (Ostlund, 2022). Organic production has grown rapidly in the last several years due to premium prices in the marketplace, among other factors (Strik et al., 2017).

Organic blueberry production in the PNW has many challenges, including diseases like mummy berry caused by the fungus *Monilinia vaccinii-corymbosi* (Reade) Honey. Mummy berry is characterized by floral and shoot blight, discoloration, and shriveling of berries before harvest (Alvarez Osorio et al., 2022). Management of mummy berry using traditional organic materials has not been effective (Pscheidt and Ocamb, 2023). Monetary losses under organic production commonly amount to 70%–85% of the production value (Florence and Pscheidt, 2017). Management recommendations for organic growers should focus on reducing overwintering inocula. This includes the removal of diseased flowers, shoots, and pseudosclerotia (commonly known as mummies) during the growing season. Once the pseudosclerotia fall to the ground, growers are advised to disrupt apothecia formation in spring through burial with mulch (Florence and Pscheidt, 2017), burning (Lambert, 1990), and raking or cultivation (Pscheidt and Ocamb, 2023).

Organic growers have developed other interesting ideas for mummy berry management after understanding the disease cycle (Pscheidt, personal communication). Some of the ideas include forgoing the yield for one year or switching to conventional strategies using synthetic fungicides, which are more effective. The intent with conventional fungicides was to transition back to organic production after a significant reduction in the primary inoculum after a year of good disease control. These ideas assume that pseudosclerotia only survive to produce apothecia for one year. Although these tactics may reduce the inoculum in the following year, the survival of pseudosclerotia from previous years would negate this benefit.

There has been speculation and some informal qualitative observations suggesting that pseudosclerotia can survive for multiple years. Ngugi et al. (2002) stated that pseudosclerotia left on the soil surface or buried at a shallow depth would disintegrate before being able to germinate; however, a small proportion may remain viable for periods exceeding 1 year. In low-bush blueberry (*V. angustifolium* Ait.) production, apothecia can be produced up to 2 years after the pseudosclerotia falls to the ground (Thompson and Annis, 2014). Based on observations of mummy corrals (Florence and Pscheidt, 2017), pseudosclerotia located within a highbush blueberry planting can survive and produce apothecia two years after contact with the soil surface.

These observations were made under blueberry bushes with a history of mummy berry. Although it seems clear that pseudosclerotia can produce inoculum for more than one year after falling to the ground, the number of years they can survive and their abundance is unknown. If pseudosclerotia can germinate after multiple years, then apothecia emerging from the ground in any year may have originated from pseudosclerotia from 2 or more years prior. Our objective was to evaluate the development of apothecia from a known number of pseudosclerotia over multiple years in a location without a history of blueberry production.

## 2 Materials and methods

**Figure 1:**
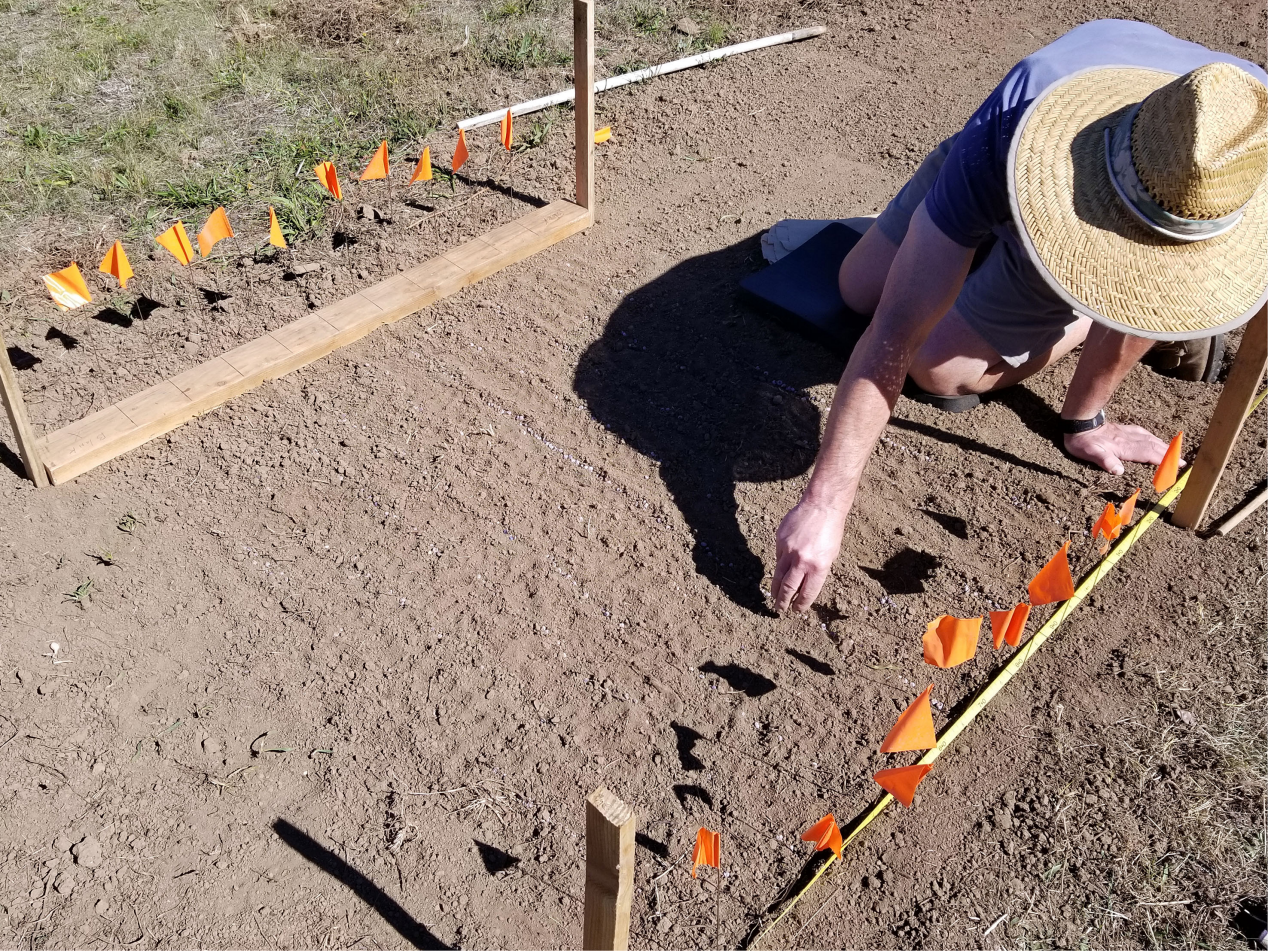
Placement of pseudosclerotia in rows prior to lightly covering with soil on 26 Sept 2018.

**Figure 2:**
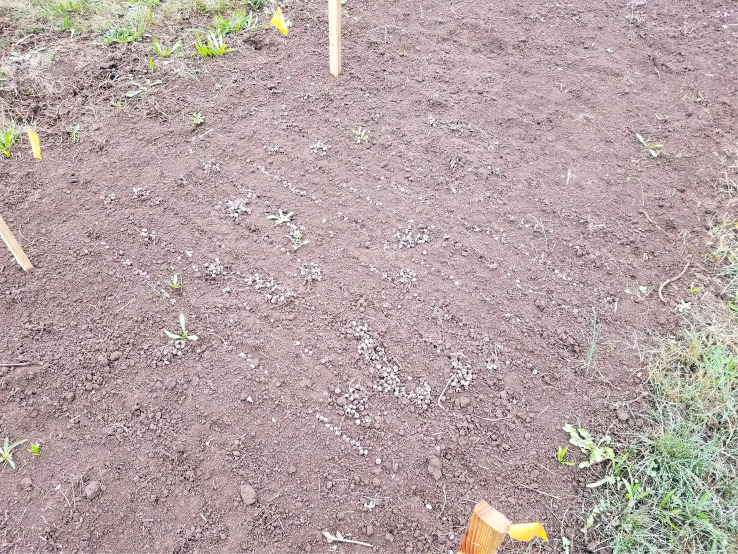
Arrangement of pseudosclerotia 24 hours later, 27 Sept 2018.

A 2 × 16.8 m (7 × 55 ft) section of land at the Botany and Plant Pathology Field Laboratory, Corvallis, OR, never planted with blueberries, was selected for multi-year observations of pseudosclerotia germination. Plots were tilled with a Troy-built rototiller before the pseudosclerotia distribution. Mummified fruits were collected from different blueberry bushes during the 2018 harvest season at an organic farm near Eugene, OR, and stored in a cold room. Mummified fruits were obtained on 21 Jul 2018, spread out in a thin layer and dried on newspaper. Pseudosclerotia were placed in 10 rows, 5 cm apart, with 100 pseudosclerotia each, for a total of 1,000 pseudosclerotia in a 1.5 × 1.5 m (5 × 5 ft) area, and lightly covered with soil on 26 Sept 2018 (**Figure 1**). Eight such plots were made with 0.3 m (1 ft) between plots for a total of 8,000 pseudosclerotia. Although the pseudosclerotia were placed in neat rows, many were scattered and/or grouped by foraging earthworms within 24 h (**Figure 2**).

**Figure 3:**
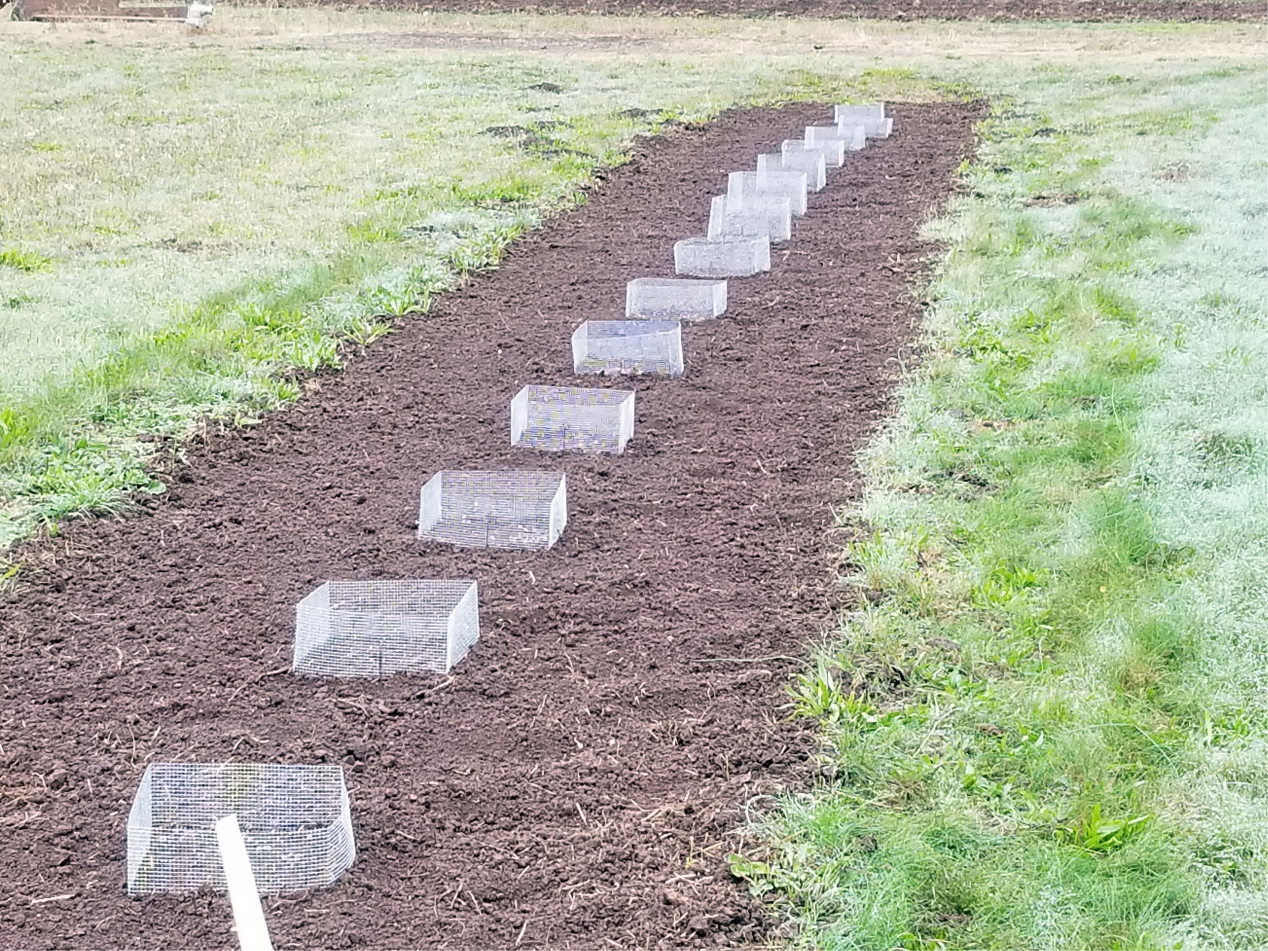
Set of square wire corrals each containing 100 pseudosclerotia.

A similar set of 152 × 152 mm (6 × 6 in) square wire corrals was also placed in a nearby 2 x 16.8 m (7 × 55 ft) section of land, each containing 100 pseudosclerotia and replicated 15 times with 0.9 m (3 ft) between plots (**Figure 3**). The temperature and rainfall were monitored using a Meter ATMOS 41 All-In-One weather station equipped with standard sensors and a ZL6 data logger. All plots were observed for pseudosclerotia and apothecia during each spring between 2019 and 2023.

Weeds were managed with herbicides and superficial burning within this area to identify emerging apothecia easily. The plots were sprayed with glyphosate (formulated as Makaze by Loveland) and the tank was mixed with aminopyralid and metsulfuron–methyl (formulated as Stinger by Corteva) on 13 Feb 2019. The plots were again sprayed with glyphosate (formulated as Makaze by Loveland); however, the tank was mixed with glufosinate-ammonium (formulated as Rely by Bayer) on 15 Oct 2019. Herbicides were applied using a “Green Thumb” hand-pump sprayer for weed management. Weed growth and plant debris that were difficult to manage with these herbicides were lightly burned away on 17 Feb 2021 and 10 Mar 2021. The plots were again sprayed with glyphosate (formulated as Mad Dog 5.4, Loveland), and the tank was mixed with glufosinate-ammonium (formulated as Forfeit, Loveland) on 26 May and 8 Sept 2022.

## 3 Results

### 3.1 Spring 2019

Rainfall for the dormant season (Oct 2018 to Mar 2019) was 24.6 cm below the 115-year average, but temperatures were average. April precipitation was 8.6 cm above normal, leading to localized flooding from 9 to 11 Apr 2019 in other parts of the farm but not in the “mummy gardens.”

Black pseudosclerotia were observed from Feb to Apr 2019 in the mummy gardens. One pseudosclerotia was at germination (as defined by Florence and Pscheidt, 2016) and two had apothecia, each in separate plots, were first observed on 8 Apr. Five apothecia were observed on 12 Apr, mostly in a single plot, and another two apothecia were observed on 15 Apr, but none were observed on 22 Apr for an approximate 7-day primary infection period. The maximum number of apothecia in any plot during the observation period was four (0.4%), and the minimum was 1.

Across the farm (400m to the southeast) in a field of Berkeley blueberries, pseudosclerotia were at germination on 18 Mar, at emergence on 25 Mar, a few were at sporulation on 29 Mar, and apothecia were also observed on 5 Apr, but not on 8 or 12 Apr for an approximate 8-day primary infection period. Very few apothecia were observed in this block compared with the previous growing seasons.

### 3.2 Spring 2020

**Figure 4:**
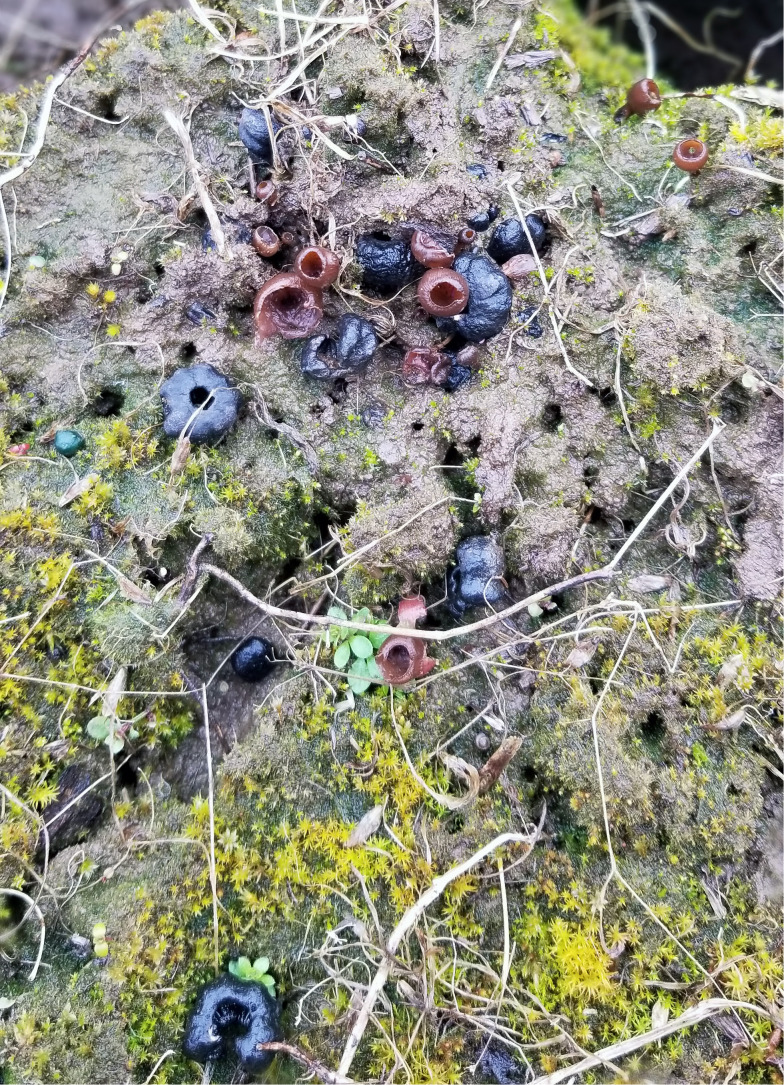
Several black pseudosclerotia on the soil surface along with brown to tan apothecia in various stages of development, March 2020.

**Figure 5:**
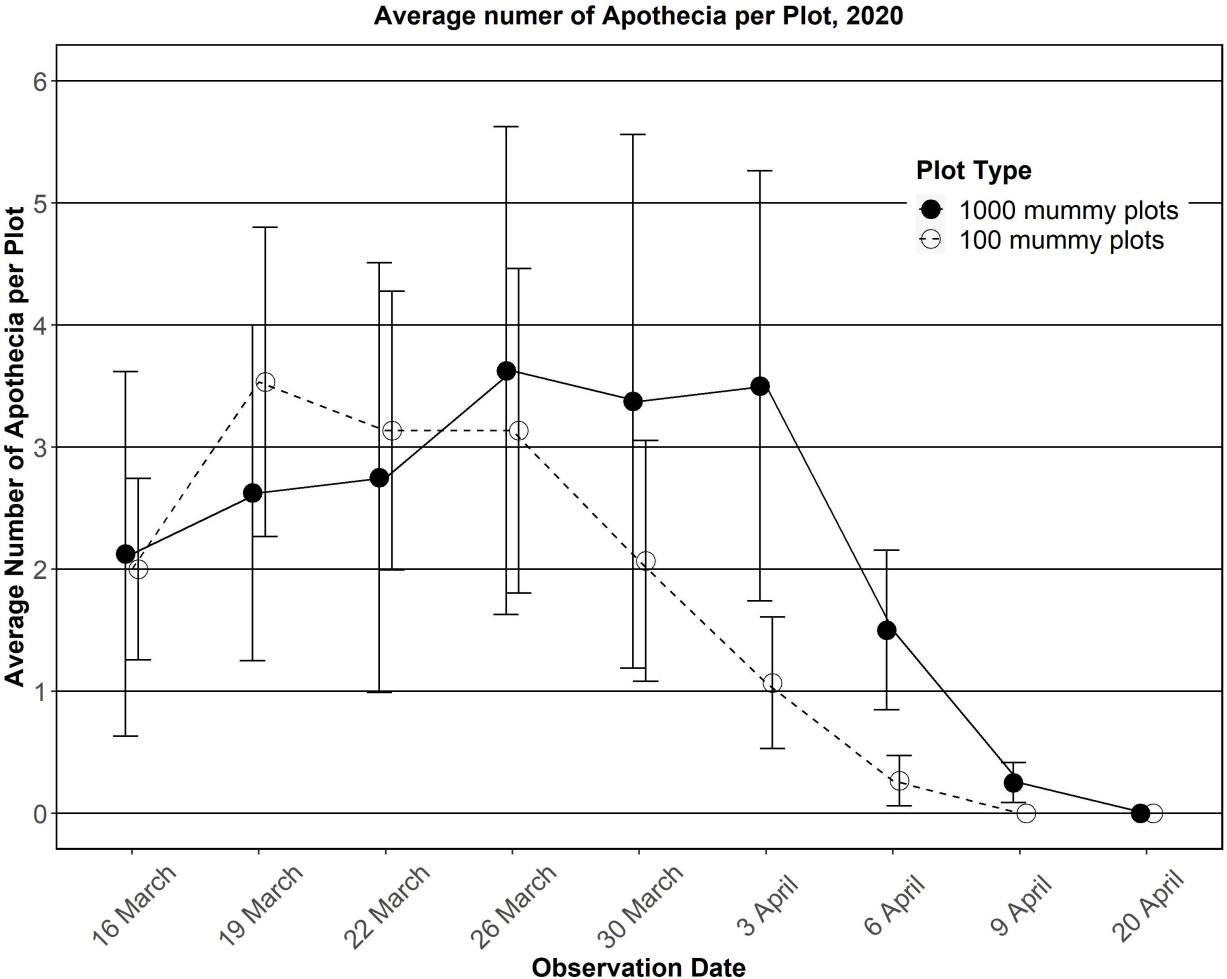
Average number of apothecia observed per plot in 2020.

Rainfall during the dormant and growing season (Oct 2019 to Sep 2020) was almost half the 116-year average. Black pseudosclerotia were partially buried by pocket gopher activity in three of the 1,000 pseudosclerotia plots and thus were not easily seen. All the other 1,000 pseudosclerotia plots had many easily observable pieces of partial or whole pseudosclerotia (**Figure 4**). Apothecia were first observed on 16 Mar 2020 and continued to appear until 9 Apr, for a 24-day infection period (**Figure 5**). The maximum number of apothecia in any plot during any observation period was 18 (1.8%), and the minimum was 0 (**Figure 5**). On 26 Mar, the average number of apothecia was 3.6 with a standard error of 2. The 100 pseudosclerotia plots showed a similar number of apothecia over a similar period (**Figure 5**).

Across the farm, in the field of Berkeley blueberries, pseudosclerotia were at germination on 2 Mar, and apothecia were observed on 25 Mar 2020, but fewer than in previous growing seasons (data not shown).

### 3.3 Spring 2021

**Figure 6:**
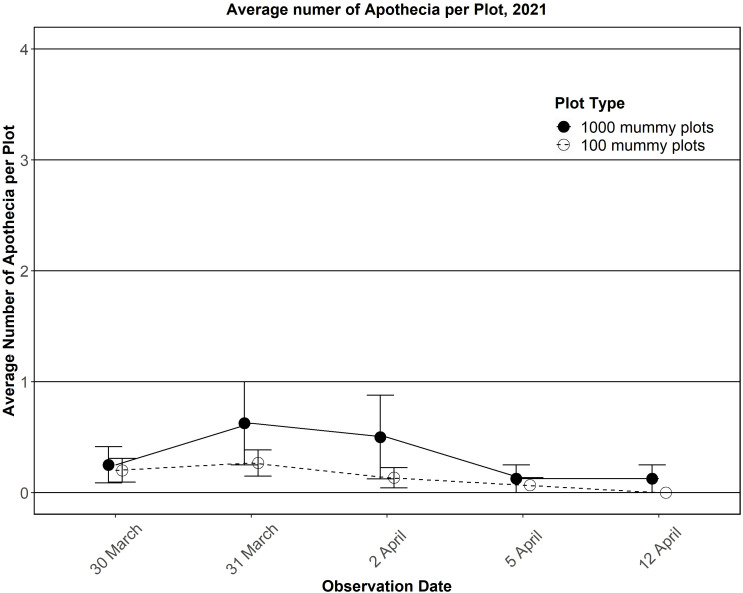
Average number of apothecia observed per plot in 2021.

Rainfall during the dormant season (Oct 2020 to Mar 2021) was close to normal; however, spring rainfall (Mar to Jun 2021) was the second lowest ever recorded. Plots in the mummy gardens contained many easily observable pieces of partial or whole pseudosclerotia. Apothecia were first observed on 29 Mar 2021 and continued to appear until 12 Apr for a 14-day infection period (**Figure 6**). The maximum number of apothecia in any plot during any observation period was 3 (0.3%), and the minimum was 0. The 100 pseudosclerotia plots showed a similar number of apothecia over a similar period (**Figure 6**).

Across the farm, in the field of Berkeley blueberries, pseudosclerotia were at germination on 22 Feb and emergence on 8 Mar. A few apothecia were observed on 29 Mar 2021, but very few compared to the prior growing seasons.

### 3.4 Spring 2022

Rainfall during the dormant season (Oct 2021 to Mar 2022) was 13.7 cm below average, but spring weather conditions (Mar to Jun 2022) were very wet, resulting in the second wettest spring on record. Unlike previous years, pieces of partial or whole pseudosclerotia were not easily observed. Of the thousands initially placed here, only one pseudosclerotium was found. In addition, despite observations every few days, no apothecia were observed in the spring of 2022. Heavy algal growth on the soil surface was observed in most plots.

### 3.5 Spring 2023

Rainfall during the dormant season (Oct 2022 to Mar 2023) was 12.2 cm below average, but spring weather conditions (Mar to May 2022) were cooler than normal. Only two apothecia were observed on 10 Apr 2023. It is hypothesized that these apothecia were approximately 2–4 days old, based on the poor condition of the cups. Apothecia were not observed one week later.

Across the farm in the field of Berkeley blueberries, pseudosclerotia were at differentiation on 27 Mar. An apothecium was observed on 9 Apr 2023 and another on 24 Apr for an approximate 15-day primary infection period.

## 4 Discussion

Pseudosclerotia of *Monilinia vaccinii-corymbosi* survived, germinated, and produced apothecia five years after being placed on the field soil. Very few of the original 9,500 pseudosclerotia from all plots and corrals produced apothecia each season. Although pieces of partial or whole pseudosclerotia were not observed after 3 years, buried pseudosclerotia could still produce apothecia.

Unexpectedly, the number of apothecia found was low, especially in the first year after pseudosclerotia placement. There may be a wide variety of reasons for the limited apothecial development. The activity of invertebrates (earthworms) and vertebrates (gopher) may have buried many pseudosclerotia below the 2.5 cm depth of field soil that apothecia have been shown to be able to emerge through (Milholland, 1974).

Herbicides have been known to affect the germination and development of apothecia (Cox and Scherm, 2001). Diuron or simazine applied during stipe development has been shown to prevent apothecia development or result in malformed apothecia. Diuron, simazine, and pre-emergent herbicides were avoided and the herbicides used were applied in the fall or dormant seasons before stipe emergence. However, the use of herbicides may have inhibited the development of apothecia. In addition, burning in low-bush blueberry fields has been associated with lower mummy berry severity (Lambert, 1990). Although flaming was used in 2021 to remove dry vegetation, the application was not extensive or for a significant period of time. We do not believe that the technique affected pseudosclerotial germination, but this cannot be ruled out.

Using a field without a history of blueberry production prevents mummy berry contamination from previously infected blueberry crops. However, the lack of a blueberry stimulus may have hindered the pseudosclerotial germination. Floral extracts from susceptible cultivars increase *Colletotrichum fioriniae* (ripe rot) appressoria formation and the infection of blueberry and cranberry fruits (Waller et al., 2018; Waller et al., 2020). Although such feedback has not been shown for the mummy berry disease, exudates from blueberry roots, leaves, or debris may help stimulate pseudosclerotia germination.

Knowing that a pseudosclerotial “seed bank” exists under blueberry bushes impacts mummy berry management. Tactics such as foregoing yield for one year or going conventional for a short time, and switching back to organic will not successfully eliminate the primary inoculum. Growers that focus on the removal of pseudosclerotia at and after harvest, combined with the disruption of developing apothecia in the spring, as their primary management tactic, will have to continue these tactics for several years before they begin to have an effect on the epidemic. If fungicide resistance develops in *M. vaccinii-corymbosi* (Alvarez Osorio et al., 2022), it may persist for many years.

## 5 Summary

The development of apothecia from *M. vaccinii-corymbosi* pseudosclerotia was evaluated over multiple years at a location without a history of blueberry production. In 2018, 9,500 pseudosclerotia were placed on field soil in replicated plots and corrals. The pseudosclerotia were able to survive, germinate, and produce apothecia 5 years after their placement. Very few pseudosclerotia produced apothecia in any year, varying from 0 to 18 at any given time. Pieces of partial or whole pseudosclerotia were observed for up to three years after placement. The pseudosclerotial “seed bank” under blueberry bushes, as demonstrated in this study, necessitates the implementation of mummy berry cultural management tactics for several years.

## Data availability statement

The raw data supporting the conclusions of this article will be made available by the authors, without undue reservation.

## Author contributions

BW prepared the figures. JP wrote the first draft of the manuscript. JP and BW contributed to the manuscript revision and read and approved the submitted version. All authors contributed to the article and approved the submitted version.
